# The Sri Lankan twin registry biobank: South Asia's first twin biobank

**DOI:** 10.1017/gheg.2020.4

**Published:** 2020-09-09

**Authors:** Kaushalya Jayaweera, Lakshan Warnakula, Lasith Dissanayake, Anushka Adikari, Sisira Siribaddana, Helena M. S. Zavos, Fruhling Rijsdijk, Patricia A. Zunszain, Carmine M. Pariante, Nick Glozier, Matthew Hotopf, Athula Sumathipala

**Affiliations:** 1 Institute for Research and Development in Health and Social Care, Colombo, Sri Lanka; 2 Department of Medicine, University of Rajarata, Mihintale, Sri Lanka; 3 Department of Psychology, Institute of Psychiatry, Psychology & Neuroscience, King's College London, London, UK; 4 Social Genetic and Developmental Research Centre, Institute of Psychiatry, Psychology & Neuroscience, King's College London, London, UK; 5 Stress, Psychiatry and Immunology Laboratory, Institute of Psychiatry, Psychology & Neuroscience, King's College London, London, UK; 6 Brain and Mind Centre, University of Sydney, Sydney, Australia; 7 Department of Psychological Medicine, Institute of Psychiatry, Psychology, and Neuroscience, King's College London, London, UK; 8 South London and Maudsley NHS Foundation Trust, London, UK; 9 Research Institute for Primary Care & Health Sciences, Faculty of Medicine & Health Sciences, Keele University, Keele, UK

**Keywords:** Biobank, DNA, population based, serum, Sri Lanka, twin research

## Abstract

**Introduction:**

Biobanks are a valuable resource for creating advancements in science through cutting-edge omics research. Twin research methods allow us to understand the degree to which genetics and environmental factors contribute to health outcomes.

**Methods:**

The Sri Lankan Twin Registry biobank (SLTR-b) was established in 2015 as part of Colombo Twin and Singleton Follow-up Study. Venous blood and urine were collected from twins and comparative sample of singletons for clinical investigations and biobanking.

**Results:**

The SLTR-b currently houses 3369 DNA and serum samples. Biobank specimens are linked to longitudinal questionnaire data, clinical investigations, anthropometric measurements, and other data.

**Discussion:**

The SLTR-b aims to address gaps in health and genetics research. It will provide opportunities for academic collaborations, local and international, and capacity building of future research leaders in twin and omics research. This paper provides a cohort profile of the SLTR-b and its linked data, and an overview of the strategies used for biobanking.

## Introduction

Twin research methods are genetically sensitive designs which allow researchers to understand the degree to which genetics and environmental factors contribute to variation (individual differences) in variables such as health outcomes. In addition, twin studies allow for associations between traits (disorders) to be quantified in parts due to shared genes and shared environmental factors.

## Materials and methods

The Sri Lankan Twin Registry was established in 1997, and is the first ever twin register in South Asia [1]. It is still one of the few large-scale functional population-based twin registries in the developing world [2]. In 2007, twins from the registry and a comparative sample of singletons (non-twins) were recruited from Colombo district to conduct a cross-sectional study on mental health titled ‘The Colombo Twin and Singleton Study’ (COTASS 1) [3]. The study was a collaboration between the Institute for Research and Development, Colombo, Sri Lanka (IRD), the Institute of Psychiatry, Psychology and Neuroscience, Kings College London, UK, and Brain and Mind Centre, University of Sydney, Australia. COTASS 1 aimed to bridge a gap in genetic research on mental health in a low- and middle-income country by exploring the genetic and environmental factors involved in common mental disorders in the Sri Lankan population.

The same cohort was revisited in 2012 to conduct a multi-component study on mental health, metabolic syndrome factors, and other health and well-being variables (the Colombo Twin and Singleton follow-up Study – COTASS 2) [4]. The Sri Lankan Twin Registry biobank (SLTR-b) was established as a component of COTASS 2 with the aim to foster innovative epidemiological research. Participant recruitment lasted until the end of December 2015 for all components.

### Setting

The COTASS cohort is based in the Colombo district which is a multi-ethnic and multicultural city [5]. Although being the country's capital, it is also the administrative and economic centre of the country. It is the most highly populated district in Sri Lanka with 2.4 million people comprising 11.4% of the country's population. Colombo has a mix of urban and rural areas with the majority being urban. The district is heavily urbanised and more westernised than other areas of Sri Lanka. The population is characterised by great socio-economic diversity in terms of education, employment, and occupational social class.

COTASS 2 comprised of three main study components: surveys, anthropometric measures, and biospecimen collection for clinical investigations and biobanking. This report is an overview of the outcomes, and proceedings of collecting, processing, and storing biospecimen data for the SLTR-b.

### Minimising pre-analytical errors

Pre-analytical errors to ensure accurate laboratory results were minimised through careful collection and handling of biospecimens [6]. Similar care was taken with biospecimens collected for long-term storage for biobanking such that they would be usable for future downstream processes. A 4-h time frame for biospecimen collection and transportation back to the laboratory was maintained to reduce serum protein concentrations change over time [7, 8]. Pre-printed stickers with participant's unique identification number were used to label collected samples thereby avoiding unreadable or incorrectly written identification numbers. Standard operating procedures for biospecimen collection, storage, and participant preparation instructions for biospecimen collection were developed and optimised during the pilot phase. These minimised variations as biospecimens were collected from multiple sites which included clinical collection centres and house visits.

As infection, inflammatory processes, and medications may influence the outcome of certain blood investigations [9], participants were excluded from the clinical investigations’ component if they (1) were on long-term medication which could interfere with the parameters under the study (e.g. steroids or other immunomodulatory agents); or (2) suffered from a comorbid inflammatory disease (e.g. chronic infections and autoimmune diseases). Pregnant participants and those suffering from an acute ailment or infection were revisited after delivery or recovery of illness, respectively.

### Biospecimen collection

Overnight fasting blood and first morning mid-stream urine was collected contemporaneously from a single participant to measure a variety of clinical investigations and for biobanking (see Table 1 for details). Blood was collected using a vacuum extraction tube system (Becton Dickinson & Co Vacutainer System). Table 1 provides details of the collection order, preservative type, and volumes. Clinical investigations were outsourced to a private hospital in Colombo, housing an accredited laboratory.

**Table 1.** COTASS 2 collected sample types, collection priority, volume, and purpose

Order of collection	Type	Volume (ml)	Purpose
	**Fasting blood**		
1	EDTA	2.5	Glycated haemoglobin
2	Clot activator (SST)	3	Lipid profile, SGOT^a^, serum insulin, serum creatinine, hs-CRP^b^
3	Clot activator (SST)	5	Serum isolation for biobanking
4	Fluoride	2	Fasting blood sugar
5	EDTA	6	DNA isolation for biobanking
	**First morning urine**		
	Sterile specimen bottle	10	Urine creatinine, urine micro albumin, urine albumin-creatinine ratio

a Serum glutamic oxaloacetic transaminase.

b Highly sensitive c reactive protein.

Participants were given the options of (1) visiting the IRD; (2) visiting the main collection centre of the private hospital or one of their collection centres; or (3) have a research team visit their homes for biospecimen collection. Home visits were chosen by the majority of participants. The two teams conducting field biospecimen collection included a phlebotomist and two research assistants; one of whom had a medical background and the other in charge of driving and navigation. A single team was able to visit between 10 and 15 participants on a single day in the morning. Participants were grouped into clusters based on their geographic proximity to each other. Therefore, each participant's overnight fasting time remained between 9 and 12 h. Passive containers with plastic transport racks stored inside rigifoam boxes layered with coolant gel packs were used for specimen transportation. Biospecimens collected from the satellite centres as well as at the IRD and by the research teams were transported directly to the main laboratory of the private hospital within the strict 4-h time frame.

### Processing of biospecimens for clinical investigations and biobanking

Blood and urine samples intended for clinical investigations were processed by staff at the main laboratory. Blood and urine not used in clinical investigations was discarded. The 5 ml clot activator tubes were centrifuged and the separated serum (approximately 1.5–2 ml) was immediately divided into two equal aliquots. These were transferred into screw-top cryogenic vials using micropipettes and stored in a −80 °C freezer until further testing.

Guidelines for biobanking standards have not been developed for Sri Lanka as yet. Therefore, we adopted several international guidelines to ensure integrity and viability of DNA samples. DNA extraction from the whole blood samples was carried out at IRD's genetics laboratory using a Flexi Gene 250 DNA Purification Kit (Qiagen, Germany) under sterile conditions. DNA of each participant was divided into two equal aliquots and stored in 1.4 ml cryo-vials. The paired aliquots were stored in separate cryo-boxes and transferred back to the −80 °C freezer.

The quality/purity of the extracted DNA were controlled by measuring the absorbance (*A*) of every sample at 260, 280, and 230 nm spectrophotometrically and determining the *A*_260_/*A*_280_ and *A*_260_/*A*_230_ ratios. For samples of good quality, *A*_260_/*A*_280_ is expected to lie in the range of 1.6–2.1. Lower values may indicate protein contamination whereas higher values may indicate a high content of RNA. At SLTR-b, the average ratio *A*_260_/*A*_280_ in general is found to be 1.8 (±0.2). The *A*_260_/*A*_230_ ratio was used as a secondary measure of DNA purity. Expected *A*_260_/*A*_230_ values for pure DNA are commonly within the range between 2.0 and 2.2. If the ratio is lower than expected, it may indicate the presence of contaminants such as proteins, Guanidine HCL (used for DNA extraction), EDTA, salts, or phenols. At SLTR-b, the average *A*_260_/*A*_230_ ratio is found to be 2.0 (±0.2). Integrity of DNA samples was assessed by agarose gel electrophoresis.

### Clinical investigation reports

Original reports for clinical investigations were sent to participants whereas a digital copy was securely stored at IRD. We provided a cover letter which matched the participant's name to the identification number in the investigation reports. Body mass index (BMI), waist circumference, and blood pressure were included in this letter.

### Biospecimen storage

Biobanking samples were labelled using cryogenic labels and stored in a large capacity −80 °C Forma 88600V freezer (Thermo Fisher Scientific, USA). A strictly controlled temperature is maintained to avoid unnecessary freeze–thaw cycles which may affect the levels of serum biomarkers [10] or yield of extracted genomic DNA [11]. During the biospecimen collection period, the freezer was initially stored in the main laboratory, which had backup generators and 24-h surveillance. The freezer was locked, and contents only accessible to authorised personnel of the COTASS 2 team.

In 2017, the −80 °C freezer and its content were transferred to the IRD's genetics laboratory while ensuring reliable power backup and greater security. A generator powered backup system was installed at IRD premises to ensure an uninterrupted power supply. It was coupled with a GSM power failure alarming system to alert the function of the power backup system using mobile short messenger service notifications.

### Ethics and governance

COTASS 2 received ethical approval from the Faculty of Medical Sciences University of Sri Jayewardenepura, Sri Lanka Ethical Review Committee (reference number: 596/11), and the Psychiatry, Nursing & Midwifery Research Ethics Subcommittee, King's College London, UK (reference number: PNM/10/11-124). Data collection procedures and storage of biospecimens were in accordance with ethical standards of both institutional review boards, the ethical policies and practices of the IRD [12], and with the Helsinki Declaration of 1975. Informed written consent from all participants were obtained using two sections in the same form; one for each of the components of the main study including clinical investigations, and another section specifically designed for long-term storage of serum and DNA. Participants were over 18 years of age and could opt out of any or all of the components of COTASS 2. Participants were able to consent to (1) clinical investigations only, (2) DNA and serum storage for biobanking only, or (3) both clinical investigations and biobanking. Respondents visiting the IRD for biospecimen collection were provided with a simple meal to break their fast after sample collection. The main laboratory of the private hospital was provided with only the participants’ unique identification number, age, and gender to maintain anonymity and confidentiality. Participants with clinical investigations indicating any departures from the norm were advised to seek medical attention from the health centre nearest to them. The wide range of clinical investigations were a great benefit to many, who were attending diabetic or cardiovascular disease clinics. Ethical considerations for stored biospecimens are mentioned in the discussion section.

## Results

Biospecimen collection was completed by the end of December 2014. DNA extractions were completed in February 2016, following which quality and integrity analysis of these samples continued for another 6 months and concluded in August 2016. Samples for biobanking and clinical investigations were collected from 3357 and 3464 participants, respectively. The high-participation rate was due to the availability of house visits and high standard of participant engagement by the FRAs.

There is a slight preponderance of females in the cohort. Participant ages range from 19 to 91 years (mean age: 42.81 years). The large majority of participants were of Sinhalese ethnicity (92.94%). There was a high participation from urban areas, and many respondents were unemployed and non-manual or skilled manual workers. One percent of respondents had no education, and the many participants (46%) had been schooled up to General Certificate of Education (Ordinary Level). The COTASS 2 sociodemographic details of participants are detailed in Table 2. Seventy-four percent of the participants are individual twins, as one singleton was recruited for every pair of twins in COTASS 1. The total overall number of individual twins for DNA and serum are 2485 and 2565 respectively, and 27% are opposite gender twins. Zygosity characteristics are presented in Table 3.

**Table 2.** Sociodemographic characteristics of COTASS 2 participants in the SLTR-b

	Biobanking component *N* = 3357		Clinical investigations *N* = 3464	
Characteristics	*n*	%	*n*	%
Twin status				
Singleton	872	25.88	899	25.95
Twin (individual)	2485	74.02	2565	74.05
Gender				
Female	1895	56.45	1958	56.52
Male	1462	43.55	1506	43.48
Age				
19–29 years	719	21.42	742	21.42
30–39 years	863	25.71	898	25.92
40–49 years	718	21.39	741	21.39
50–59 years	580	17.28	596	17.21
60–69 years	314	9.35	320	9.24
70+ years	163	4.86	167	4.82
Ethnicity^a^				
Sinhala	3093	92.94	3186	92.83
Tamil	103	3.09	106	3.09
Muslim	118	3.55	126	3.67
Other	14	0.42	14	0.41
Urbanicity				
Rural	1339	39.89	1392	40.18
Urban	2018	60.11	2072	59.82
Employment^a^				
Unemployed, retired or student	1432	43.33	1479	43.39
Elementary occupations	285	8.62	288	8.45
Non-manual or skilled manual workers	1335	40.39	1378	40.42
Managers or professionals	253	7.66	264	7.74
Education level^a^				
No education	38	1.15	39	1.14
Grade 1–5	235	7.09	236	6.91
Grade 6 up to G.C.E. (O/L)^b^	1530	46.18	1571	45.99
Completed G.C.E. (O/L) Exam	527	15.91	548	16.04
Up to or passed G.C.E. (A/L)^c^ Exam	758	22.88	788	23.07
University or higher	225	6.79	234	6.85

a Contains missing data.

b General Certificate of Education (ordinary level).

c General Certificate of Education G.C.E. (advanced level).

**Table 3.** Zygosity characteristics of twins (complete and incomplete pairs) of the SLTR-b

Zygosity	DNA (%)	Serum *n* (%)
Monozygotic females	605 (24.35)	621 (24.21)
Monozygotic males	467 (18.79)	483 (18.83)
Dizygotic females	405 (16.30)	421 (16.41)
Dizygotic males	316 (12.72)	325 (12.67)
Dizygotic opposite gender	692 (27.85)	715 (27.88)
Total	2485 (100.00)	2565 (100.00)

Anthropometric measures, blood pressure, clinical investigations, and longitudinal survey data are linked to the SLTR-b. Other data include heart rate variability data and actigraphy data from two sub-studies within COTASS 2. Table 4 provides details of all linked data, and Table 5 provides a list of selected COTASS 2 mental and physical health characteristics of participants in the SLTR-b and associations or mean differences between genders.

**Table 4.** Types of data linked to the SLTR-b from the longitudinal COTASS study

Data type	COTASS 1 (2006–2007)	COTASS 2 (2012–2015)	DNA (*N*)	Serum (*N*)
Surveys				
Sociodemographic data	Sociodemographic questionnaire	Sociodemographic questionnaire	3357	3464
Depression	Lifetime version of WHO-CIDI^a^ Section E	12-month version of section E of the WHO-CIDI	3329	3433
		Beck Depression Inventory II	3312	3416
Post-traumatic stress disorder	WHO-CIDI Section K	17 items PTSD Civilian Checklist	3326	3430
Anxiety	WHO-CIDI Section D	Generalised Anxiety Disorder 7 item	3323	3427
Somatic symptoms	Bradford Somatic Inventory	Bradford Somatic Inventory	3357	3464
Fatigue	Chalder Fatigue Scale	Chalder Fatigue Scale	3357	3464
Tobacco use	WHO-CIDI Section B	Tobacco Use questionnaire	3357	3464
Alcohol use	WHO-CIDI Section J	Alcohol Use Disorders Identification Test	3326	3430
Life events	The list of threatening experiences	The list of threatening experiences	3324	3428
Health and well-being	Short Form-36 Health Survey	Short Form 36 Health Survey	3329	3442
Twin zygosity	Zygosity determination questionnaire	Zygosity determination questionnaire	2485	2565
Closeness of twins	Not done	Closeness of twins questionnaire	2438	2515
Environmental stressors	Childhood experience of care and abuse questionnaire^b^	Not done	3362	3362
Suicidal ideations	Suicidal ideations questionnaire	Not done	3362	3362
Traumatic events	War and tsunami questionnaire	Not done	3362	3362
Social support	Not done	Multi-dimensional support scale questionnaire	3323	3427
Eating disorders	Not done	Eating disorders questionnaire^b^	3329	3433
Dietary habits	Not done	Diet and frequency of food groups^c^	3338	3442
Physical illnesses	Not done	A checklist of physical illnesses	3327	3431
Physical activity	Not done	International Physical Activity Questionnaire	3312	3416
Sleep	Not done	Pittsburgh sleep quality index	3329	3433
Other				
Biochemistry	Not done	Clinical urine tests	3352	3459
		Clinical blood tests	3357	3464
Anthropometric measurements	Not done	Height, sitting height, weight, waist circumference	3330	3437
Cardiovascular assessment	Not done	Resting blood pressure	3333	3440
		Heart rate variability data^d^ ϕ♂	256	266
Sleep and activity monitoring	Not done	Actigraphy^e^ ϕ	214	220

a World Health Organisation – Composite International Diagnostic Interview.

b The Dutch Eating Behaviour Questionnaire and the Three-Factor Eating Questionnaire were adopted for this eating disorders questionnaire.

c A questionnaire to measure the frequency of consumption of a comprehensive list of food groups in Sri Lanka.

d Recordings done on a Schiller Medilog AR12 Plus Holter Recorder at 1000 Hz.

e Data collected using Phillips Actiwatch Spectrum.

ϕ Data from twins only.

♂ Data from males only.

**Table 5.** Selected COTASS 2 health characteristics linked to the DNA in the SLTR-b and associations or mean differences between genders

	Females	Males	χ^2^/*t*-test *p* value
	%/mean (*n*)	%/mean (*n*)	
*Mental health*			
Depressive symptoms			
None to mild	55.28% (1670)	44.72% (1351)	<0.001
Moderate to severe	68.87% (208)	31.13% (94)	
PTSD			
No	56.19% (1785)	43.81% (1392)	0.019
Symptomatic	65.63% (105)	34.38% (55)	
Anxiety			
None to mild	54.93% (1598)	45.07% (1311)	<0.001
Moderate to severe	68.24% (290)	31.76% (135)	
*Substance use*			
Smoking status			
Doesn't smoke	64.87% (1898)	35.13% (1028)	<0.001
Current smoker	1.35% (6)	98.65% (437)	
Alcohol use			
No misuse	64.94% (1889)	35.06% (1020)	<0.001
Misuse	0.23% (1)	99.77% (427)	
*Blood pressure*			
Systolic (mmHg)	114 (1888)	119 (1455)	<0.001
Diastolic (mmHg)	75 (1888)	77 (1455)	0.0002
*Anthropometry*			
Height (cm)	151.46 (1887)	165.41 (1453)	<0.001
Weight (kg)	56.19 (1888)	62.82 (1453)	<0.001
Waist Circumference (cm)	90.99 (1886)	86.59 (1452)	<0.001
BMI (kg/m^2^)	24.46 (1886)	22.89 (1453)	<0.001
*Biochemistry*			
Serum creatinine (mg/dl)	0.74 (1904)	0.98 (1465)	<0.001
Total cholesterol (mg/dl)	200.90 (1904)	198.61 (1465)	0.1053
Low-density lipoprotein (mg/dl)	127.31 (1904)	123.02 (1465)	0.0005
Very low-density lipoprotein (mg/dl)	22.42 (1904)	28.69 (1465)	<0.001
High-density lipoprotein (mg/dl)	51.18 (1904)	46.89 (1465)	<0.001
Triglycerides (mg/dl)	113.06 (1904)	147.36 (1465)	<0.001
Total cholesterol/HDL ratio	4.03 (1904)	4.36 (1465)	<0.001
SGOT (U/l)	22.73 (1904)	29.43 (1465)	<0.001
Serum insulin (uU/ml)	13.45 (1902)	13.07 (1465)	0.5618
Ultra-sensitive c-reactive protein (mg/l)	3.60 (1904)	2.95 (1465)	0.0018
Fasting blood sugar (mg/dl)	108.49 (1904)	106.29 (1465)	0.1268
HbA1c (%)	6.05 (1904)	5.90 (1465)	0.0019
Urine creatinine (mg/dl)	84.12 (1900)	128.23 (1464)	<0.001
Urine microalbumin (mg/l)	17.59 (1900)	22.35 (1464)	0.0195
Urine microalbumin/creatinine (mg of albumin/g)	34.63 (1900)	30.95 (1464)	0.6048

## Discussion

‘Research infrastructure is an indispensable component of research enterprise’ [13]. New knowledge and innovation can only emerge from high-quality and accessible research infrastructures which include data resources, archives, and biobanks [14]. Biobanks are a valuable resource for creating advancements in science through cutting-edge omics research. However, there is a scarcity of biobanks in the South Asian region. This may be due to a multitude of reasons including limitations in infrastructure, capacity, and sociological or ethical issues less common in Western settings. In addition, the lack of an overarching research culture in developing countries may be a systematic barrier, resulting in limited new knowledge output and capacity building within low- and middle-income countries. One of the main aims of setting up the IRD was to overcome this by helping to create an overarching research culture in Sri Lanka, and providing resources and infrastructure to local and international scientists.

COTASS 1 data have been shared for higher degree programmes locally and internationally. Until now, some COTASS 2 data have already been pooled into the CODAtwins project which is the largest international effort utilising the classical twin design [15]. Similarly, the newly established biobank is open for collaborative studies with academic institutions after research protocols have been accepted by the SLTR-b steering and ethics committees.

Human biospecimen collection for research and genetic studies are extremely sensitive matters especially in the developing world [16–19]. Unethical collection and exportation of biospecimen during events such as the 2004 tsunami [20] have made Sri Lankans apprehensive about biospecimen collection for research purposes. Ethical appropriateness varies between settings; understanding attitudes, views and beliefs of the community and stakeholders related to genomics research and biobanking is important [18]. We recently completed a qualitative study on public understanding of genomic medicine and research among SLTR-b participants [21]. Community engagement generates public trust which is of paramount importance. The IRD was awarded grants from Wellcome Trust, UK (2009) and the Medical Research Council, UK (2018) to engage twins in research through cultural activities.

Ethics forms a crucial cornerstone in the SLTR-b. Beliefs and values of our local communities played a major role in the method and types of biospecimens collected and stored, all of which must be taken into consideration. The IRD established policies and guidelines for the Sri Lankan Twin Registry [12] which conform to accepted international standards in genomic studies. The IRD's policy on human biospecimens is to analyse the data within a national institution or laboratory if the necessary infrastructure and technology exists. To address these concerns, the IRD's genetics laboratory was established during COTASS 1 as a strategic accomplishment from an ethical and infrastructure point of view [22], thereby avoiding the necessity for exportation biospecimens abroad. Broad consent was not obtained from COTASS 2 participants for biobanking. Therefore, any new studies using SLTR-b specimens will require new ethical approval and consent from study participants as well.

The main strengths of the SLTR-b include the use of the twin design with a large population-based collection of DNA and serum from twins and singletons. Biospecimens are linked to a variety of clinical biomarkers, physical measures, survey data on common mental disorders, other data, and a comprehensive set of confounders. We have been able to cover a wide age range, and the younger age groups provide an opportunity to understand disease development over future follow-up studies of COTASS. Weaknesses include the lack of clinical data from COTASS 1, and limited ethnic diversity. The cohort size of the SLTR-b is relatively small in comparison with other international biobanks such as the UK Biobank [23]. However, the SLTR-b was a component within COTASS 2, and therefore we believe we have done justice to the limited available funding. The biobank has limited geographic coverage (Colombo district only) and may limit generalisability to other populations.

In 2019, we received a Medical Research Council, UK grant to conduct a pilot study using the COTASS cohort (and their children) to disentangle genetic and environmental components of intergenerational risk transmission of nutritional choices on risk factors for cardio vascular disease. This is the first of its kind in a low- and middle-income country, and will use a children-of-twins design to identify the extent to which the association between parental and offspring nutrition is due to genetic and environmental transmission. The full grant is being applied for in 2020 and we shall be using DNA from the SLTR-b for GWAS to answer our research questions as well.

To conclude, the SLTR-b is a unique data-rich resource comprising genomic DNA and serum of twins and a matched sample of non-twins from Colombo, Sri Lanka. We have shown that large-scale research infrastructure development is possible in low- and middle-income countries through North–South collaborations. This is the ‘first of its kind’ biobank establishment in South Asia which will allow us to answer complex research questions, whereas aiming to address gaps in health and genetics research. It will provide opportunities for further academic collaborations local and internationally, and capacity building of future research leaders in twin and omics research. The future goal of the IRD is to create a nationally representative repository of biospecimens for use in innovative epidemiological research providing new insights into diseases burdening the region.
